# Retrospective Study of the Prevalence, Histopathology, Therapy, and Survival Time of Neoplastic Disease in Fish

**DOI:** 10.3390/ani14030464

**Published:** 2024-01-31

**Authors:** Emma Ferraro, Scott H. Harrison, Elizabeth Duke, Brigid Troan, Amy Boddy, Lisa M. Abegglen, Tara M. Harrison

**Affiliations:** 1 College of Veterinary Medicine, North Carolina State University, 1060 William Moore Drive, Raleigh, NC 27607, USA; emmaferraro97@gmail.com (E.F.); ecgraebe@ncsu.edu (E.D.); brigid@troan.org (B.T.); 2 Department of Biology, North Carolina Agricultural and Technical State University, 1601 E. Market St., Greensboro, NC 27411, USA; scotth@ncat.edu; 3 Exotic Species Cancer Research Alliance, College of Veterinary Medicine, 1060 William Moore Drive, Raleigh, NC 27607, USA; amyboddy@ucsb.edu (A.B.); lisa.abegglen@hci.utah.edu (L.M.A.); 4 North Carolina Zoological Park, 4401 Zoo Parkway, Asheboro, NC 27205, USA; 5 Department of Anthropology, University of California, Santa Barbara, Santa Barbara, CA 93106, USA; 6 Division of Pediatric Hematology/Oncology, Department of Pediatrics, University of Utah, Salt Lake City, UT 84112, USA

**Keywords:** cancer, neoplasia, fish, shark, sarcoma, carcionoma, zoo, aquarium, pathology, histopathology

## Abstract

**Simple Summary:**

The purpose of this study is to evaluate veterinary records of fish diagnosed with cancer to determine the most common presentation of this disease and the efficacy of various treatments. Currently, there are no comprehensive analyses of cancer across all fish species, and this study serves to aid veterinary clinicians in the management of these patients. Fish serve an important role in society as companion animals, educational ambassadors, and research models, and advances in their standard of care benefit veterinary medicine and several other industries.

**Abstract:**

This study evaluated neoplasia in fish using medical records from zoos, aquariums, and exotic animal veterinarians. The parameters evaluated included geographic location, habitat type, signalment, anatomic location of neoplasia, type of neoplasia as confirmed with histologic examination, survival time, and treatments provided for each patient. These data were entered into the Exotic Species Cancer Research Alliance (ESCRA) database. Out of 455 cases from across the United States and England, most animals submitted were from zoologic parks or aquariums (62.9%), followed by private ownership (1.5%). The percent of female (19.3%) and male (17.8%) patients were similar, and the mean age at the time of diagnosis was 99.45 months, with a range of 12 to 300 months. The species with the highest neoplasia prevalence was koi (18.5%), followed by goldfish (10.8%). The eye was the most commonly reported site for a primary neoplasm (8.4%), and the most prevalent diagnosis across all organ systems was soft tissue sarcoma (26.2%). Only 13 patients in this study (2.9%) received any form of treatment, with a mean survival time of 8.85 months post-treatment. These data demonstrate that while information related to clinical therapy of cancer in fish species is lacking, surgical excision of tumors in fish, when feasible for the patient and client, may improve patient outcomes.

## 1. Introduction

In recent years, the ownership of pet fish in the United States has been reported to reach over 76,000 animals across over 10,000 households, and these numbers only climbed during the SARS-CoV-2 pandemic [1,2]. Given the increasing need for veterinary management of captive fish in both household and zoological collections, it is imperative to understand the presentation and treatment of their diseases, including neoplasia. Neoplasia is a particularly interesting category of disease to study in fish, as there are limited studies and reports assessing its presentation and pathogenesis in these species.

This study defines neoplasia, used synonymously with cancer, as the uncontrolled growth of cells within a living organism, which disrupts the normal tissue architecture. Tumor refers to the resulting gross or histologic mass of neoplastic cells. Benign tumors, including lipomas, fibromas, and seminomas, may cause local pathology such as irritation or compression of local tissues, but do not cause generalized or systemic disease. Malignant tumors, including sarcomas and carcinomas, may cause systemic disease through metastasis, defined as the spread of tumors to secondary anatomic locations, or through generalized metabolic dysfunction resulting from a variety of tumor-specific mechanisms.

The Exotic Species Cancer Research Alliance (ESCRA) was founded to improve veterinary cancer understanding and therapy through research collaboration between human and exotic animal oncology experts. This mission of cancer research is carried out in part with the collection of case reports, which are submitted to the organization’s Exotic Tumor Database (ETD). Data are submitted via an open-source, online form that requests case information including but not limited to species, cancer diagnosis, and treatments provided. These cases are submitted to ESCRA from collaborating zoological parks, aquariums, wildlife rescue organizations, pathology services, and veterinary clinics. Data from these sources are collected through research agreements with the contributing institution.

Using the ETD, cases of confirmed neoplasia in fish were screened for meeting the criteria of this study and submitted to ESCRA. The resulting data were then extracted from the ETD and analyzed for clinical and reported pathological interpretation.

## 2. Materials and Methods

ESCRA-collaborating institutions submitted patient records, including pathology reports, via email, and these records were screened for the following criteria to determine their relevance to the present study: (1) a patient belonging to one of the taxonomic groups of hagfish, lampreys, cartilaginous fish, and bony fish; (2) the species of the patient was stated in the report; and (3) a histologic diagnosis of neoplasia, excluding xanthomas, collagenomas, hematomas, cysts, granulomas, hamartoma, hyperplasia, keratoacanthoma, or polyps. Qualifying cases were individually read and entered into the ETD. The specific data evaluated for this report included the geographic location of the submitting institution, habitat type, signalment, anatomic location of cancer, type of cancer as confirmed with histopathologic examination, survival time, and treatments provided for each patient. Data from each of these categories were analyzed with a standard percentage of the total number of cases.

Survival time by species, sex, tumor location, histologic tumor type, status of being benign or malignant, and treatment type were analyzed using IBM Corp., released 2019, IBM SPSS Statistics for Windows, Version 26.0. Armonk, NY, USA [3], and R statistical computing software (version 3.6.1; R Core Team 2020. R: A language and environment for statistical computing. R Foundation for Statistical Computing, Vienna, Austria, https://www.R-project.org/, accessed 18 January 2022) [4]. Frequencies, survival curve analysis, and boosting analyses were used to evaluate the data. For survival curve analysis, Kaplan–Meier survival curves and log-rank tests were generated with the survminer package (R package version 0.4.1, https://CRAN.R-project.org/package=survminer, accessed 18 January 2022) [5] and survival package (R package version 2.38, https://CRAN.R-project.org/package=survival, accessed 18 January 2022) [6]. For the statistical method of boosting, the mboost package (Model-based boosting, R package version 2.6.0, https://CRAN.R-project.org/package=mboost, accessed 18 January 2022) [7] was used. Survival time in months was the outcome variable. Fish without a known cause of death, or who were lost to follow-up, were excluded from the outcome analysis. The modeled effects of variables were compared to a set of 500 null model distributions of effects generated from modeling performed with permutated outcomes, with *p* < 0.05 being the threshold of significance regarding the tails of the null model distribution (two-sided hypothesis), similar in method to Mayr et al. [8].

## 3. Results

A total of 455 patients with confirmed neoplasia, representing 178 species of bony and cartilaginous fish, were included in this study. These patients were predominantly submitted from zoos, parks, and aquariums (62.9%) primarily located in the eastern United States, with some submissions from the American Midwest, American Southwest, England, and Canada. Reports from pet fish (1.5%) were also submitted from veterinary institutions. Cancer diagnoses were made either at the time of biopsy or necropsy between the years 2001 and 2022.

The most prevalent species with reported neoplasia was koi (*Cyprinus rubrofuscus*) at 18.5% of all cases, followed by goldfish (*Carassius auratus*) at 12.5% (Table 1). These two species are particularly well-represented due to their popularity as pets and ornamental fish in public collections. The mean age at presentation was 99.45 months or 8.25 years, which translates to a variety of life stages depending on species. In koi, the most prevalent species in this study, this age aligns with young adulthood (average lifespan of 40 years) [9]. There was no significant difference in the reported sex of patients with neoplasia in this study.

**Figure 1:**
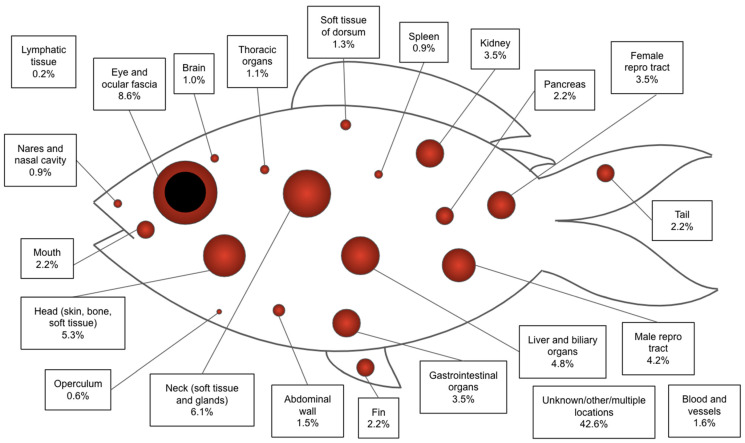
Relative frequency of anatomic location of histologically confirmed primary neoplasms in fish species.

**Figure 2:**
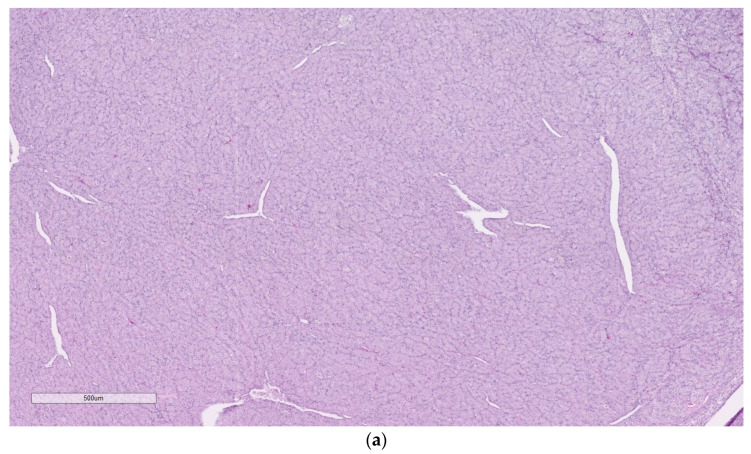

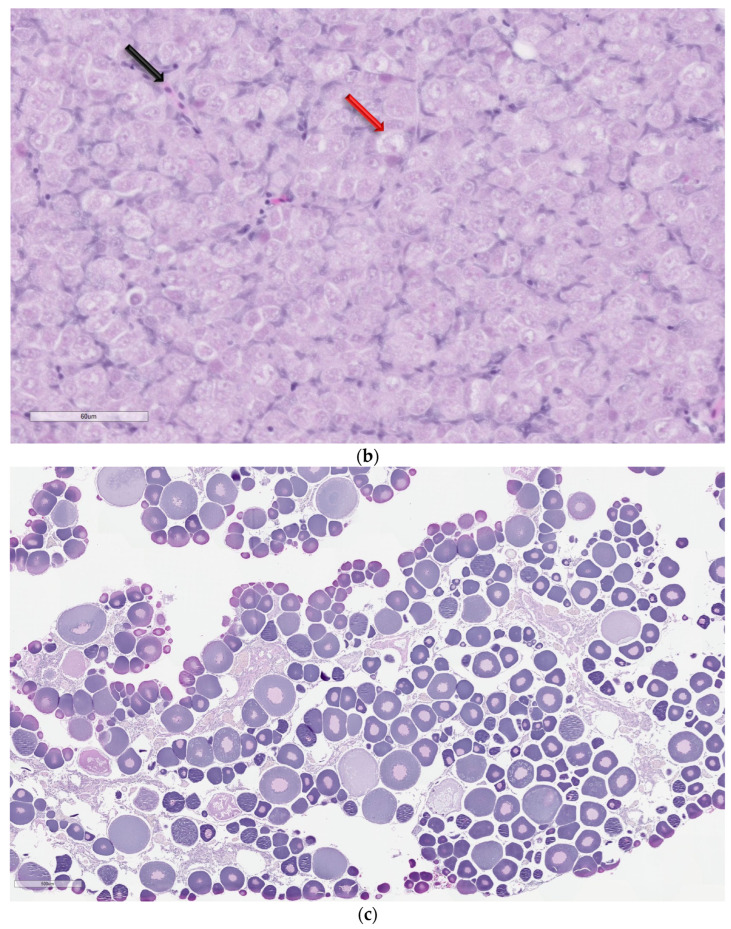

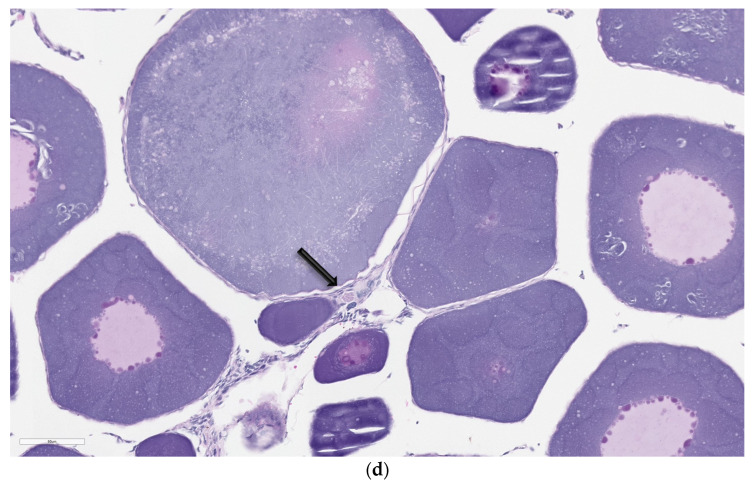
(**a**,**b**). Dysgerminoma from a chub. The neoplasm is comprised of sheets of dysplastic oogonia (indicated with a red arrow), displaced by occasional interstitium (indicated with a black arrow). Hematoxylin and eosin. (**c**,**d**) Normal control histology of a stage 1 (early development) ovary in a chub (oogonia indicated with a black arrow). Hematoxylin and eosin.

**Figure 3:**
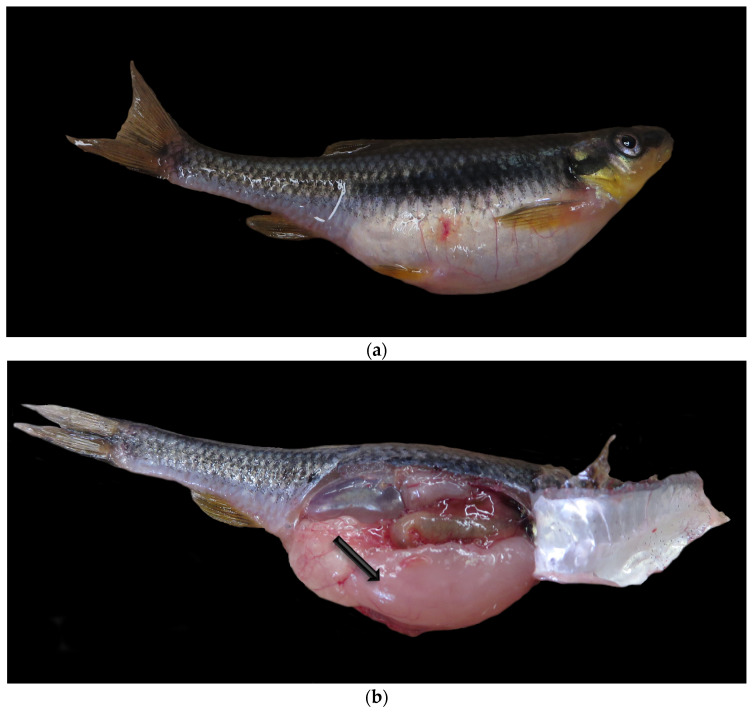

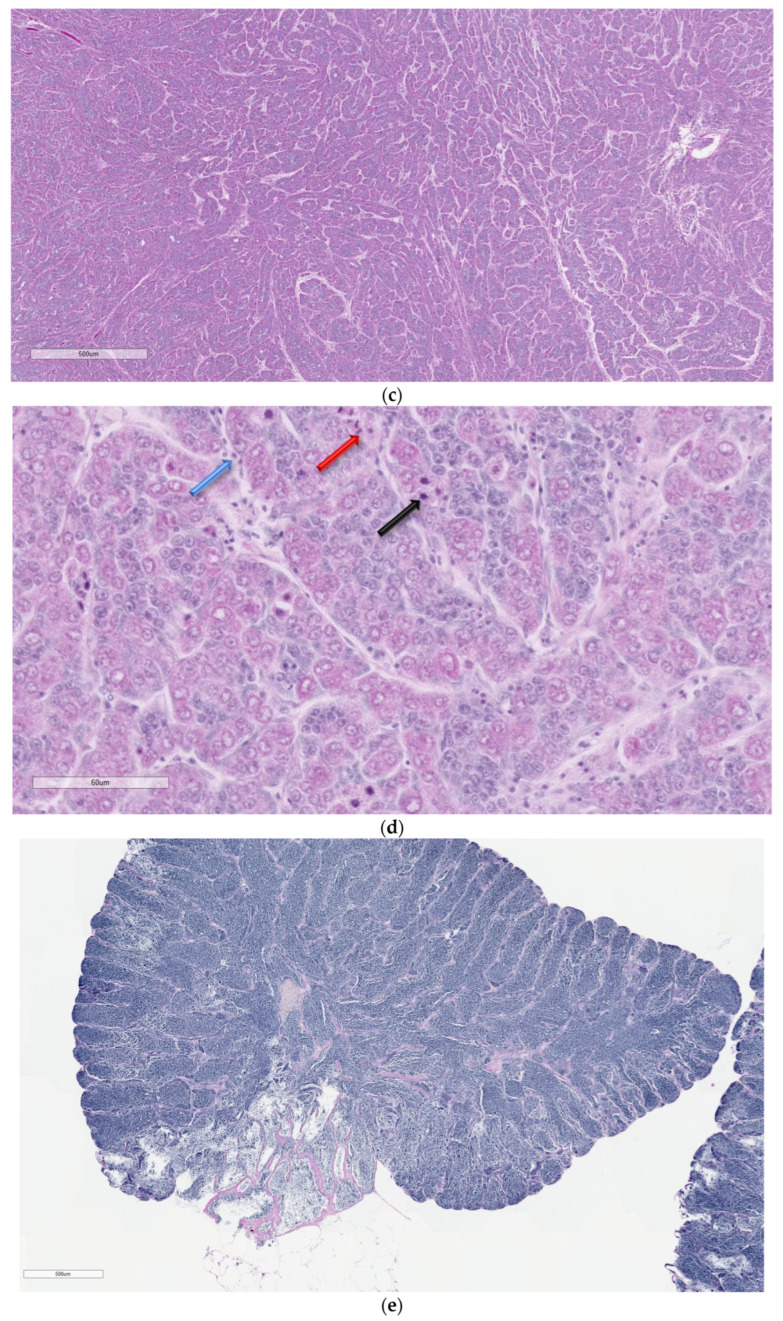

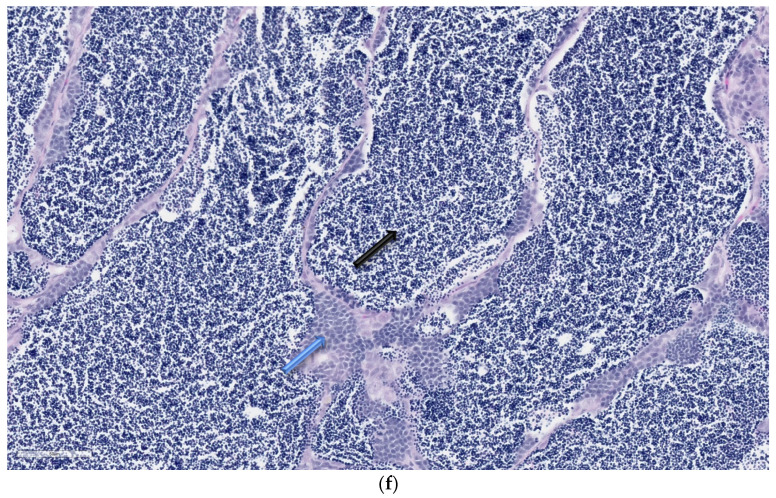
(**a**,**b**) Gross image of seminoma (indicated with a black arrow) from a chub. (**c**,**d**) Histology of seminoma from a blacknose dace. The seminiferous tubules are markedly expanded by sheets of germ cells at varying stages of development (indicated with a black arrow) with a degenerative, compressed germinal epithelium (indicated with a blue arrow). There is a high mitotic index (mitotic figures indicated with a red arrow). Hematoxylin and eosin. This case was submitted outside the study window. (**e**,**f**) Normal control histology of a stage 3 (late spermatogenic) testis from a golden shiner. Normal spermatozoa (indicated with a black arrow) are supported by a germinal epithelium (indicated with a blue arrow). Hematoxylin and eosin.

**Figure 4:**
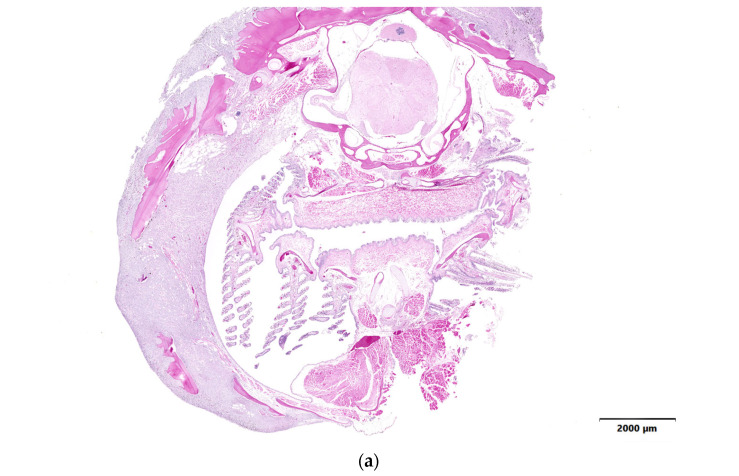

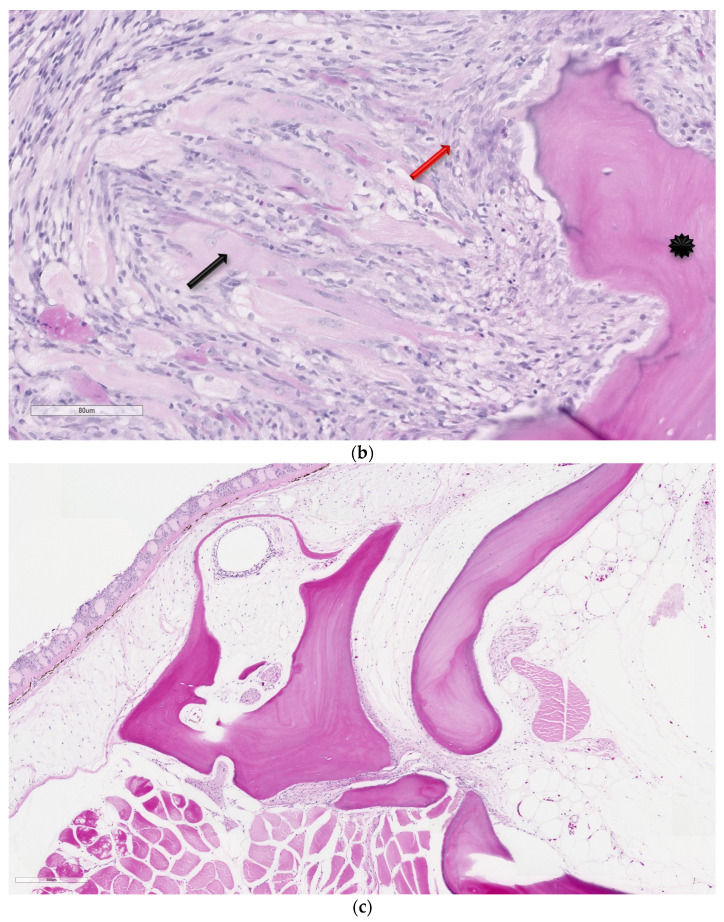

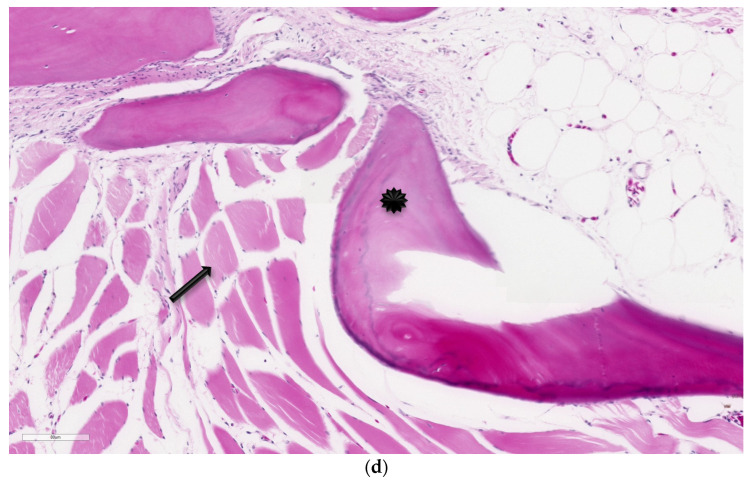
(**a**,**b**) Soft tissue sarcoma from the operculum and head of a blacknose dace. The soft tissue around the face is multifocally effaced and expanded by interlacing streams and bundles of neoplastic spindle cells (indicated with a red arrow), entrapping muscle (indicated with a black arrow), and bone (indicated with a star). Hematoxylin and eosin. This case was submitted outside the study window. (**c**,**d**) Normal internal control of soft tissue from the contralateral side of the same blacknose dace. Normal muscle (indicated with a black arrow) and bone (indicated with a star).

**Figure 5:**
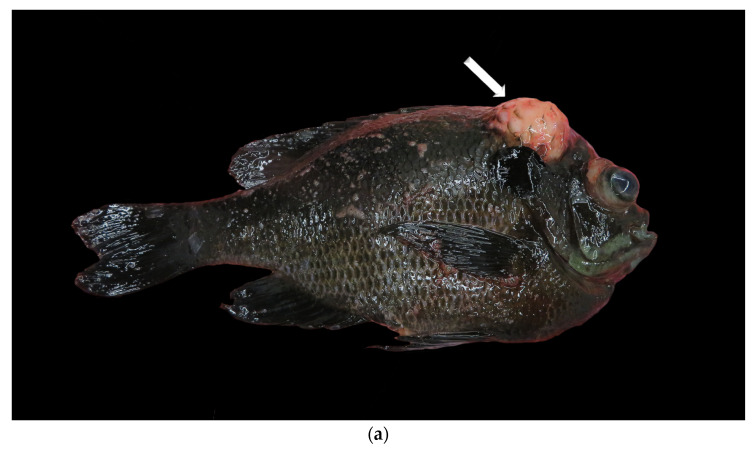

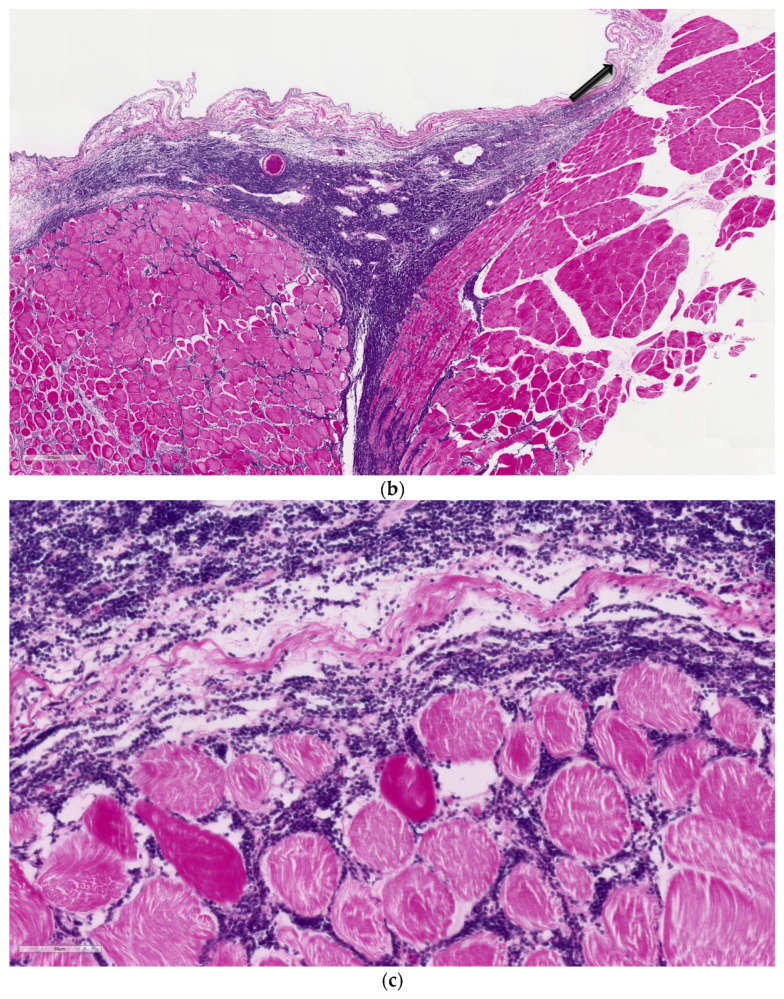
(**a**) Gross image of lymphoma (indicated by arrow) from a bluegill. This case was submitted outside the study window. (**b**,**c**) Histology of lymphoma from the same bluegill. The skin, subcutis, and musculature are expanded by infiltrative lymphocytes (indicated with a white arrow). Internal normal control of skin (indicated with a black arrow). Hematoxylin and eosin.

The most common histologic diagnosis at 26.4% of cases was soft tissue sarcoma, which was reported in several anatomic regions throughout the fish, mostly on externally visible structures (Figure 1, Figure 2, Figure 3, Figure 4 and Figure 5, Table 2). These malignant tumors were diagnosed in a variety of tissue sites, and while grading schemes have been established for domestic species [10], histologic grades were not assigned for many of the cases in this study due to the absence of references for fish. At 8.4%, the eye was the most common single site of presentation across all tumor types (with an additional 0.2% of cases originating from the fascia or adnexa surrounding the eye), excluding unspecified or varied locations in the skin and subcutis (Figure 1). Among soft tissue sarcoma affecting koi and goldfish, the most prevalent tumor type in the two most prevalent species (*n* = 65), 80% of the tumors were either in multiple sites or the location was not reported, 7.7% were in the eye, 4.6% were in the tail, 3.1% were in the soft tissues of the head, 1.5% were in the oral cavity, 1.5% were in the fin, and 1.5% were in the gastrointestinal tract.

Only 2.86% of all primary tumors in this study were treated (13 cases); a mean post-treatment survival time of 8.85 months was reported. Treatments included chemotherapy (15.4% of treated tumors), supportive care (7.7%), or more commonly, surgical excision (69.2%) (Table 3). One patient received treatment, but the treatment type was not specified (7.7%).

Patient sex, histologic tumor type, status of benign and metastasis, treatment type, and tumor location all had no significant effect on survival time.

- Species frequency

**Table 1:** Frequency and percentage of the 20 most prevalent fish species that had histologically confirmed neoplasia.

Species Common Name (Scientific Name)	Frequency (Percentage)
Koi (*Cyprinus rubrofuscus*)	84 (18.5)
*Goldfish* spp. (*Carassius auratus*)	57 (12.5)
Bluegill (*Lepomis macrochirus*)	9 (2)
Foureyed fish (*Anableps* spp.)	7 (1.5)
Polar or arctic cod ((*Boreogadus saida*)	7 (1.5)
Unspecified African cichlid (*Cichlidae* spp.)	5 (1.1)
Betta (*Betta splendens*)	5 (1.1)
Largemouth bass (*Micropterus salmonides*)	5 (1.1)
Red bellied piranha (*Pygocentrus nattereri*)	5 (1.1)
Angelfish (*Pterophyllum* spp.)	4 (0.9)
Bumblebee cichlid (*Pseudotropheus crabro*)	4 (0.9)
Creek chub (*Semotilus atromaculatus*)	4 (0.9)
Lined seahorse (*Hippocampus erectus*)	4 (0.9)
Arapaima (*Arapaima gigas*)	3 (0.7)
Black tetra (*Gymnocorymbus ternetzi*)	3 (0.7)
Brook trout (*Salvelinus fontinalis*)	3 (0.7)
Clown loach (*Chromobotia macracanthus*)	3 (0.7)
Cownose ray (*Rhinoptera bonasus*)	3 (0.7)
Flamefish (*Apogon maculatus*)	3 (0.7)
Grubby sculpin (*Myoxocephalus aenaeus*)	3 (0.7)

- Anatomic tumor location

- Histologic diagnosis

**Table 2:** The twenty most prevalent histologically confirmed neoplasia diagnoses of fish species.

Histologic Tumor Diagnosis	Frequency (Percentage)	Class Affected (Number of Cases with Diagnosis)	Species Affected Common Name (Scientific Name) (Number of Cases with Diagnosis)
Soft tissue sarcoma	120 (26.4)	Ray-finned fish (118), cartilaginous fish (1), unknown class (1)	Koi (*Cyprinus rubrofuscus*) (36), goldfish (*Carassius auratus*) (33), cichlid (*Cichlidae* spp.) (4), pufferfish (*Tetraodontidae* spp.) (3), scorpionfish (*Scorpaenidae* spp.) (3), seahorse (*Hippocampus* spp.) (3), bluegill (*Lepomis macrochirus*) (2), foureyed fish (*Anableps* spp.) (2), leaf fish (*Monocirrhus polyacanthus*) (2), sunfish (*Centrarchidae* spp.) (2), pupfish (*Cyprinodontidae* spp.) (2), alewife (*Alosa pseudoharengus*) (1), angelfish (*Pterophyllum* spp.) (1), arapaima (*Arapaima gigas*) (1), Atlantic halibut (*Hippoglossus hippoglossus*) (1), Atlantic spadefish (*Chaetodipterus faber*) (1), cardinalfish (*Apogonidae* spp.) (1), brook trout (*Salvelinus fontinalis*) (1), crevalle jack (*Caranx hippos*) (1), false herring (*Harengula clupeola*) (1), flounder (*Paralichthys dentatus*) (1), gray snapper (*Lutjanus griseus*) (1), goodeid (*Goodeidae* spp.) (1), killifish (*Fundulus majalis*) (1), largemouth bass (*Micropterus salmonides*) (1), Pacific sanddab (*Citharichthys sordidus*) (1), piranha (*Pygocentrus nattereri*) (1), prickly leatherjacket (*Chaetodermis penicilligerus*) (1), pumpkinseed fish (*Lepomis gibbosus*) (1), rainbowfish (*Melanotaeniidae* spp.) (1), rainbow trout (*Oncorhynchus mykiss*) (1), Rockfish (*Sebastidae* spp.) (1), spotfin hatchetfish (*Thoracocharax stellatus*) (1), stingray (*Myliobatoidei* spp.) (1), threespot headstander (*Pseudanos trimaculatus*) (1), walleye (*Sander vitreus*) (1), warmouth (*Lepomis gulosus*) (1), yellow perch (*Perca flavescens*) (1), unknown species (1)
Hemolymphatic/round cell (lymphoma/leukemia)	48 (10.5)	Ray-finned fish (40), cartilaginous fish (6), unknown class (2)	Koi (*Cyprinus rubrofuscus*) (7), cardinalfish (*Apogonidae* spp.) (4), cichlid (*Cichlidae* spp.) (4), cownose ray (*Rhinoptera bonasus*) (3), bluegill (*Lepomis macrochirus*) (2), brook trout (*Salvelinus fontinalis*) (2), electric eel (*Electrophorus electricus*) (2), pike characin (*Acestrorhynchus microlepis*) (2), pipefish (*Syngnathinae* spp.) (2), sunfish (*Centrarchidae* spp.) (2), tetra (*Characiformes* spp.) (2), archerfish (*Toxotes chatareus*) (1), carp (*Cyprinus carpio*) (1), creek chub (*Semotilus atromaculatus*) (1), goldfish (*Carassius auratus*) (1), grubby sculpin (*Myoxocephalus aenaeus*) (1), guitarfish (*Rhinobatos rhinobatos*) (1), pinktail chalceus (*Chalceus macrolepidotus*) (1), pumpkinseed fish (*Lepomis gibbosus*) (1), red drum (*Sciaenops ocellatus*) (1), stingray (*Myliobatoidei* spp.) (1), tinfoil barb (*Barbonymus schwanenfeldii*) (1), whitespotted bamboo shark (*Chiloscyllium plagiosum*) (1), wolf eel (*Anarrhichthys ocellatus*) (1), wrasse (*Labridae* spp.) (1), unknown species (2)
Chromatophoroma	35 (7.9)	Ray-finned fish (33), cartilaginous fish (2)	Goldfish (*Carassius auratus*) (9), koi (*Cyprinus rubrofuscus*) (9), betta (*Betta splendens*) (4), catfish (*Siluriformes* spp.) (2), cichlid (*Cichlidae* spp.) (2), bowfin (*Amia calva*) (1), Cardinalfish (*Apogonidae* spp.) (1), clownfish (*Amphiprion ocellaris*) (1), largemouth bass (*Micropterus salmonides*) (1), nurse shark (*Ginglymostoma cirratum*) (1), seahorse (*Hippocampus* spp.) (1), slender madtom (*Noturus exilis*) (1), southern ray (*Hypanus americanus*) (1), spindle hap (*Protomelas taeniolatus*) (1)
Thyroid carcinoma or adenocarcinoma	23 (5.1)	Ray-finned fish (22), cartilaginous fish (1)	Angelfish (*Pterophyllum* spp.) (4), grunt (*Haemulon flavolineatum*) (2), lookdown fish (*Selene vomer*) (2), rarrotfish (*Scaridae* spp.) (2), Atlantic spadefish (*Chaetodipterus faber*) (1), bamboo shark (*Chiloscyllium plagiosum*) (1), black ghost (*Apteronotus albifrons*) (1), black surfperch (*Embiotoca jacksoni*) (1), cichlid (*Cichlidae* spp.) (1), chalk bass (*Serranus tortugarum*) (1), cunner fish (*Tautogolabrus adspersus*) (1), high hat (*Pareques acuminatus*) (1), killifish (*Fundulus majalis*) (1), pupfish (*Cyprinodontidae* spp.) (1), rock beauty (*Holacanthus tricolor*) (1), rock goby (*Gobius paganellus*) (1), seahorse (*Hippocampus* spp.) (1)
Carcinoma (unspecified)	18 (4.0)	Ray-finned fish (18)	Koi (*Cyprinus rubrofuscus*) (7), goldfish (*Carassius auratus*) (2), rockfish (*Sebastidae* spp.) (2), cardinalfish (*Apogonidae* spp.) (1), cichlid (*Cichlidae* spp.) (1), clownfish (*Amphiprion ocellaris*) (1), glofish (*Danio rerio*) (1), largemouth bass (*Micropterus salmonides*) (1), monkey-face eel (*Cebidichthys violaceus*) (1), oscar (*Astronotus ocellatus*) (1)
Squamous cell carcinoma	16 (3.5)	Ray-finned fish (14), cartilaginous fish (1), unknown class (1)	Koi (*Cyprinus rubrofuscus*) (7), cod (*Gadus* spp.) (3), goldfish (*Carassius auratus*) (2), cichlid (*Cichlidae* spp.) (1), discus (*Symphysodon aequifasciata*) (1), bamboo shark (*Chiloscyllium plagiosum*) (1), unknown species (1)
Seminoma	14 (3.1)	Ray-finned fish (12), lobe-finned fish (1), unknown class (1)	Cichlid (*Cichlidae* spp.) (2), creek chub (*Semotilus atromaculatus*) (2), crested blenny (*Hypleurochilus geminatus*) (2), sunfish (*Centrarchidae* spp.) (2), koi (*Cyprinus rubrofuscus*) (1), lungfish (*Dipnoi* spp.) (1), Nile perch (*Lates niloticus*) (1), pumpkinseed fish (*Lepomis gibbosus*) (1), rainbowfish (*Melanotaeniidae* spp.) (1), unknown species (1)
Nerve sheath tumor	11 (2.4)	Ray-finned fish (11)	Goldfish (*Carassius auratus*) (2), catfish (*Siluriformes* spp.) (1), creek chub (*Semotilus atromaculats*) (1) flamefish (*Apogon maculatus*) (1), foureyed fish (*Anableps* spp.) (1), koi (1), oscar (*Astronotus ocellatus*) (1), pumpkinseed fish (*Lepomis gibbosus*) (1), tetra (*Characiformes* spp.) (1), yellow perch (*Perca flavescens*) (1)
Fibroma	10 (2.2)	Ray-finned fish (9), cartilaginous fish (1)	Koi (*Cyprinus rubrofuscus*) (2), Atlantic black-tipped shark (*Carcharhinus limbatus*) (1), cardinalfish (*Apogonidae* spp.) (1), catfish (*Siluriformes* spp.) (1), foureyed fish (*Anableps* spp.) (1), goldfish (*Carassius auratus*) (1), menhaden (*Brevoortia tyrannus*) (1), piranha (*Pygocentrus nattereri*) (1), seahorse (*Hippocampus* spp.) (1)
Hepatocellular carcinoma	8 (1.8)	Ray-finned fish (8)	Arapaima (*Arapaima gigas*) (1), arowana (*Scleropages formosus*) (1), cichlid (*Cichlidae* spp.) (1), Goatfish (*Mullidae* spp.) (1), Pacific halibut (*Hippoglossus stenolepis*) (1), riponianus (*Haplochromis riponianus*) (1), seahorse (*Hippocampus* spp.) (1), sunfish (*Centrarchidae* spp.) (1)
Papilloma	8 (1.8)	Ray-finned fish (5), cartilaginous fish (3)	Bamboo shark (*Chiloscyllium plagiosum*) (2), rainbow trout (*Oncorhynchus mykiss*) (2), common shiner (*Luxilus cornutus*) (1), foureyed fish (*Anableps* spp.) (1), leopard shark (*Triakis semifasciata*) (1), walleye (*Sander vitreus*) (1)
Lipoma	6 (1.3)	Ray-finned fish (6)	Bullhead (*Ameiurus melas*) (2), moray eel (*Muraenidae* spp.) (1), Northern spearnose poacher (*Agonopsis vulsa*) (1), piranha (*Pygocentrus nattereri*) (1), rainbowfish (*Melanotaeniidae* spp.) (1)
Ovarian carcinoma	6 (1.3)	Ray-finned fish (6)	Koi (*Cyprinus rubrofuscus*) (2), bluegill (*Lepomis macrochirus*) (1), cichlid (*Cichlidae* spp.) (1), goldfish (*Carassius auratus*) (1), tetra (*Characiformes* spp.) (1)
Myxoma	5 (1.1)	Ray-finned fish (5)	Bird wrasse (*Gomphosus varius*) (2), goldfish (*Carassius auratus*) (1), moray eel (*Muraenidae* spp.) (1), seahorse (*Hippocampus* spp.) (1)
Renal adenocarcinoma	5 (1.1)	Ray-finned fish (5)	Angelfish (*Pterophyllum* spp.) (1), grunt (*Haemulon flavolineatum*) (1), koi (*Cyprinus rubrofuscus*) (1), pearl guarami (*Trichopodus leerii*) (1), piranha (*Pygocentrus nattereri*) (1)
Retinoblastoma	5 (1.1)	Ray-finned fish (5)	Cichlid (*Cichlidae* spp.) (1), rainbowfish (*Melanotaeniidae* spp.) (1), sapphire damselfish (*Pomacentrus pavo*) (1), tinfoil barb (*Barbonymus schwanenfeldii*) (1), two-stripe whitelip (*Plectorhinchus albovittatus*) (1)
Dysgerminoma	4 (0.9)	Ray-finned fish (4)	Flounder (*Paralichthys dentatus*) (1), pipefish (*Syngnathinae* spp.) (1), seahorse (*Hippocampus* spp.) (1), scalefin fairy basslet (*Pseudanthias squamipinnis*) (1)
Intestinal adenocarcinoma	4 (0.9)	Ray-finned fish (4)	Seahorse (*Hippocampus* spp.) (2), bullhead (*Ameiurus melas*) (1), sunfish (*Centrarchidae* spp.) (1)
Pancreatic adenocarcinoma	4 (0.9)	Ray-finned fish (3), cartilaginous fish (1)	Arctic cod (*Arctogadus glacialis*) (1), leopard shark (*Triakis semifasciata*) (1), piranha (*Pygocentrus nattereri*) (1), tetra (*Characiformes* spp.) (1)
Basal cell carcinoma	3 (0.7)	Ray-finned fish (3)	Cichlid (*Cichlidae* spp.) (2), betta (*Betta splendens*) (1)

- Treatments

**Table 3:** Treatments performed in fish for primary neoplasia for all cases, with species, tumor diagnosis, sex, and average survival time reported.

Treatment Type	Frequency	Average Survival Time (Months)	Patient Sex	Species Common Name (Scientific Name) (Primary Tumor Type)
Surgery only	9	19.5	Female (3), male (2), unknown (4)	Bowfin (*Amia calva*) (chromatophoroma), arapaima (*Arapaima gigas*) (soft tissue sarcoma), prickly leatherjacket (*Chaetodermis penicilligerus*) (soft tissue sarcoma), koi (*Cyprinus rubrofuscus*) (neuroectodermal sarcoma), koi (*Cyprinus rubrofuscus*) (squamous cell carcinoma), yellow lab cichlid (*Labidochromis caeruleus*) (ocular astrocytoma), Boeseman’s rainbowfish (*Melanotaenia boesemani*) (retinoblastoma), largemouth bass (*Micropterus salmonides*) (neuronal embryonal tumor), balloonfish (*Tetraodontidae* spp.) (soft tissue sarcoma)
Chemotherapy only	2	1	Male (1), unknown (1)	Electric eel (*Electrophorus electricus*) (hemolymphatic/round cell), pumpkinseed fish (*Lepomis gibbosus*) (peripheral nerve sheath tumor)
Supportive care only	1	1	Unknown	Lined seahorse (*Hippocampus erectus)* (fibroma)
Unknown treatment	1	Unknown	Unknown	Unknown

## 4. Discussion

### 4.1. Prevalence

Despite the growing popularity of fish among pet owners, hobbyists, and parks, there are very few published reports from the current century summarizing the prevalence of tumors of various tissue origins in teleost and elasmobranch species. Currently, studies of tumors in fish are mostly limited to experimental models for human neoplasia such as swordtail-platyfish backcross hybrids and zebrafish that have been used to model melanoma [11,12,13]. One study used human-derived gene expression to induce rhabdomyoma, ocular melanoma, astrocytoma, and spindle cell sarcoma in zebrafish [14]. Despite its limited representation in the literature and the common belief that fish, especially sharks, are immune to cancer, this study confirms that the prevalence of spontaneous or naturally occurring neoplasia is found worldwide in these species.

In fact, some reported tumors are unique to fish, including those of the swim bladder and gills, due to their anatomic uniqueness to these species [11,12]. Other differences between neoplasia in fish and mammalian species include lower rates of malignancy in fish and a higher prevalence of neoplastic ectopic tissues that would normally be considered benign in mammals, with the exception of ectopic thyroid tissue [11,12]. One publication suggests that while in humans, the lungs are predisposed to neoplasia caused by environmental carcinogens due to the physiology of their respiration, in fish, the gills and skin are predisposed instead, as these tissues make direct contact with these substances [15]. It has also been proposed that cutaneous tumors may have a lower threshold for formation in fish than in humans [13]. All these differences together necessitate a thorough understanding of the prevalence, diagnosis, and treatment success of cancer in fish specifically.

Similarities between neoplasia in mammals and fish include the predilection for tissues with a normally high rate of cellular turnover, such as the gills and scales, and the histological characteristics and growth mechanisms of some of these epithelial tumors, such as melanoma [13,16]. In humans, epithelial malignancies comprise the vast majority (80–90%) of cancer diagnoses, particularly in high-turnover tissues, such as the lung, prostate, and breast [17]. In both fish and humans, this high prevalence of epithelial tumors may be somewhat biased due to their visibility when originating from external tissues. Though the specific tissue origin may differ between fish and mammals, a one-health approach to cancer research and diagnosis in both taxa may help to identify these useful trends in presentation and be used for comparative research.

### 4.2. Risk Factors

While the cause of many types of cancer in fish are largely unknown, some studies seek to investigate potential risk factors. Environmental carcinogens have been implicated in some surveys and controlled laboratory studies. Creosote and polycyclic aromatic hydrocarbons (PAHs) were shown to cause pancreatic and hepatic neoplasms in a mummichog, brown bullhead, and winter flounder [11,12,18]. Regional case reports include an English sole with a hepatocellular neoplasm due to materials found in the water of Puget Sound, eels in southern France with a high prevalence of liver and spleen tumors corresponding to a high concentration of organochlorine pesticides and PAHs in their tissues, high cadmium levels linked to liver tumors in flatfish dab in the North Sea and English Channel, and epizootic neoplasms in several fish species in a copper mining repository in Michigan [11,12,19,20,21]. Similarly, cutaneous neoplasms in wild fish in the Great Lakes have been linked to environmental contaminants, either through direct carcinogenesis or immunosuppression [18]. Experimentally, N-methyl-N-nitrosourea, nifurpirinol, and other known carcinogens were shown to cause cancer in the platyfish and croaker [18,22]. Farmed fish are at risk of feed contamination with dioxin-like compounds and aflatoxins, which may be carcinogenic [11,12].

Husbandry factors may also play a role in the formation of neoplasia. Changes in salt iodine levels in the water system of a zebrafish colony were reported to result in the formation and regression of thyroid tumors, and water chlorination has been implicated in the formation of bullhead papillomas [23,24]. A study that surveyed keepers of koi found significant correlations between neoplasia and several environmental factors including indoor pond location, large pond size, high frequency of water changes, and history of treatment with praziquantel, formalin/malachite green, or potassium permanganate; however, causation was not proven for any of these factors [25].

Internal parasitism and viruses have been linked to neoplasia in some fish species, such as viral cutaneous papillomas in the bullhead, though exact agents have not been definitively identified [11,12,18]. Retroviruses have been historically suspected, but recent studies implicate multifactorial viral etiologies [26]. It is theorized that microplastic particles in the environment may serve a role in tumor formation due to their potential to form oncogenic viral biofilms [15]. Some species and regional populations of fish may have genetic components, predisposing them to certain tumor types, such as genes that contribute to melanoma formation in zebrafish and medaka and sarcomas in the platyfish [11,12,13]. Ultraviolet light has also been implicated in melanoma formation in fish through mutagenic damage to the genome [13].

### 4.3. Diagnosis and Histopathology

Ante-mortem diagnosis of neoplasia in fish typically starts with the identification of clinical abnormalities during routine monitoring or health screening. Common initial presenting complaints reported in this study included visualization of an external mass, bulging of the eyes, coelomic distension, or behavioral changes such as aberrant swimming or inappetence. Further diagnostic techniques such as imaging or evaluation of blood parameters may support a differential diagnosis of neoplasia but do not provide a definitive diagnosis [27].

In most cases, a definitive diagnosis of neoplasia in fish is currently limited to biopsy or necropsy followed by standard histopathology. Cytologic examination of samples from impression smears or fine needle aspirates may be useful, but it is not as reliable as histopathology, and cannot be used to treat the tumor, as with an excisional biopsy [25]. Biopsy is typically only pursued in cases of cutaneous or ocular tumors due to the ease of surgical technique and good post-operative prognosis, but celiotomy or celioscopy with incisional or excisional biopsy may be attempted for internal tumors [26]. Biopsy and enucleation techniques in fish, as well as proper sample handling and submission protocols, are outlined in several veterinary resources [27,28,29].

Necropsy, while limited to patients who have a grave prognosis or die spontaneously, provides the most complete pathologic picture of cancer in an individual case, as it allows for gross and histologic evaluation of all body systems. If a board-certified veterinary pathologist, particularly one with experience in fish species, is not available to perform a necropsy, there are several resources available to guide the clinician in standard necropsy sampling protocols [11,12,27,30].

A majority of tumors are histologically diagnosed with standard hematoxylin and eosin preparation, which shows the general architecture of tissues and can be used to classify cells into broad morphologic categories. However, because of anatomic differences, many of these neoplasms, for example, sarcomas, are not well differentiated and require ancillary stains or immunohistochemistry to arrive at a specific diagnosis [31,32]. However, although useful in differentiating tumor types in domestic species, immunohistochemistry has limited use in fish due to a lack of validation and available reference reagents for these species [11,12]. When using immunohistochemistry in fish, pathologists can rely on tier 2 validation, which relies largely on internal controls [33]. Grading systems for neoplasia in non-domestic species are also limited; while comprehensive grading resources exist, these resources are predominantly for companion animal species [34] and are not reliable in fish.

### 4.4. Treatments

Treatments in this study were limited to surgical excision, chemotherapy, and supportive care, but cryotherapy and cryosurgery have also been reported with improved recovery and lower tumor recurrence when compared with surgical excision [27]. Additionally, experimental trials of oncogene inhibitor therapy show promising signs of efficacy in fish species [13].

Surgical excision is typically both therapeutic and diagnostic, following the biopsy protocols referenced above. In this study, surgery was the most prevalent treatment type and yielded a longer mean survival time than chemotherapy or supportive care alone, though these data are limited by a small sample size.

Two patients in this study received chemotherapy. An electric eel (*Electrophorus electricus*) with histologically confirmed disseminated round cell neoplasia received prednisolone 1.5 mg/kg orally once per day. This patient, however, continued to decline clinically and was euthanized after one month of treatment. A pumpkinseed fish (*Lepomis gibbosus*) was initially diagnosed with a soft tissue sarcoma, was treated with an unknown dose of an unknown anti-angiogenesis chemotherapeutic, and was ultimately euthanized after an unknown duration of treatment. Necropsy with histopathology yielded an updated diagnosis of peripheral nerve sheath tumor. In the literature, one case report describes poor response to oral lomustine, methylprednisolone, and L-asparaginase in a cownose ray (*Rhinoptera bonasus*) with multicentric lymphosarcoma. The clinicians in this case ultimately stopped this chemotherapy protocol and pursued surgical excision of the affected spleen, and the patient was euthanized intraoperatively due to lack of hemostasis [35]. Famously, a 21-year-old giant grouper (*Epinephelus lanceolatus*) at the Shedd Aquarium was reported to be treated with surgery and cisplatin for an undifferentiated mesenchymal cell tumor and survived an additional five years before dying from unrelated medical issues [36]. Other sources report local infiltration of various chemotherapeutic drugs, but survival time in these cases is not known [27].

One case in this study received supportive care alone. A lined seahorse (*Hippocampus erectus*) with a fibroma of the tail diagnosed on incisional biopsy was treated with three doses of meloxicam 0.3 mg/kg and ceftazidime 22 mg/kg every three days. The mass appeared stable until the patient died six weeks later. Other supportive care protocols used in fish species include anti-inflammatory doses of steroids [27]. Medication doses can be extrapolated from mammalian doses or found in some exotic animal formularies [27,37].

### 4.5. Limitations

The limitations of this study include the restriction of cases to those in captive management, excluding wild populations, which may affect the reported ages and exclude certain environmental factors. Also, because of the focus on captive populations, tumors of external structures are more likely to be identified by keepers and animals. The overall lack of routine internal imaging and systemic medical screening in fish patients precludes many clinicians from identifying subclinical or metastatic tumors that are not grossly visible, and internal neoplasia may be missed unless a full necropsy is performed. However, even with these limitations, valuable information can be obtained from this study that can assist veterinary clinicians in evaluating fish medical examinations as well as be aware that treatments are possible and able to be performed with early diagnosis of cancer in fish.

## 5. Conclusions

This study both emphasizes the understated prevalence of neoplasia across bony and cartilaginous fish species and offers direction for future work for the clinical and pathological workup of neoplasia in fish. Some such points for further investigation include validation of immunohistochemistry and in situ hybridization in fish for more accurate diagnoses, development of histologic grading systems in fish, and controlled experimental studies evaluating various treatments of the most common tumor types. The authors encourage clinicians and researchers in zoo, aquarium, academic, and private practice settings to continue to submit any reports of histologically confirmed neoplasia in fish to the Exotic Tumor Database [38] to aid the advancement of this research and the outcomes of fish with cancer. Additionally, the authors encourage aquatic medicine clinicians to continue to advance fish medical diagnoses with increased routine examinations and screening, which can enable additional potentially successful treatments of fish affected by cancer.

## Data Availability

All data used in this study were submitted to ESCRA through the ETD. Restrictions apply to the availability of these data. Data were obtained from the institutions acknowledged below and are available through the corresponding author with the permission of the submitting institutions.
